# Blueprint for progress: Understanding the driving forces of BIM adoption in Kingdom of Saudi Arabia (KSA) construction industry

**DOI:** 10.1371/journal.pone.0313135

**Published:** 2025-02-10

**Authors:** Muzaffar Iqbal, Irfan Ullah, Heba Abdou, Majed Alzara, Ahmed M. Yosri

**Affiliations:** 1 School of Maritime Economics and Management, Dalian Maritime University, Dalian, P. R. China; 2 Transportation Engineering College, Dalian Maritime University, Dalian, P. R. China; 3 Department of Business and Administration, ILMA University, Karachi, Pakistan; 4 Department of Interior Design, College of Engineering, Jouf University, Sakaka, Saudi Arabia; 5 Department of Civil Engineering, College of Engineering, Jouf University, Sakaka, Saudi Arabia; King Khalid University, SAUDI ARABIA

## Abstract

Building information modeling (BIM) as a virtual and digital mode of representing construction activities gained significant attention and facilitated construction projects. Nevertheless, many driving forces (DFs) trigger the adoption of BIM. Different kinds of studies have been conducted regarding the DFs of BIM adoption in developed countries. However, few studies have classified the adoption of DFs of BIM technology in developing countries such as the Kingdom of Saudi Arabia (KSA). A range of previous literature identified these DFs in a different context, but there is a need to answer two main questions. First, what DFs could influence BIM adoption in the construction sector of (KSA); second, what could be the possible framework to prioritize these DFs. Therefore, Fuzzy Delphi Methodology (FDM), Interpretive structural modeling (ISM), and MICMAC were applied to answer these questions. Study results highlight that ’Reduced cycle time of the design process’ and ’Efficient construction planning and management’, are the main DFs to BIM adoption in the construction sector of (KSA). This study is the first to employ a hybrid FDM, ISM, and MICMAC approach to evaluate BIM implementation DFs in the KSA context. This study informs policymakers and industry practitioners in (KSA) to develop targeted strategies for effective BIM adoption. This study enhances collaboration and communication among construction industry stakeholders by understanding the significant DFs and their interrelationships.

## 1. Introduction

The construction process is complex by nature, covering different phases from construction to potential destruction, and each needs vast documentation and significant information management [1]. The success of mega-construction projects relies on the collaboration and participation of experts from different organizations working toward common project objectives. To manage these intricacies and increase project productivity, building information modeling BIM has appeared as a transformative technology that grabs the attention of stakeholders across the construction industry. By incorporating BIM technology with green practices, the construction industry can significantly contribute to protecting the natural environment and developing sustainable buildings [2]. BIM technology fosters communication among project stakeholders, encourages a unified platform, and ensures interoperability, which increases communication quality among project stakeholders, supports a combined platform and provides interoperability across different business domains [3].

A study by Saka and Chan [4] highlights that stakeholders can achieve maximum advantages and sustainability in construction projects by balancing the significant variables of time, cost, and quality. This balance can be realized by strategically adopting cutting-edge technologies such as BIM. Shaqour [5] further highlights that green construction practices can be realized by incorporating sustainable technologies, with BIM being a prime example of such innovation. Olawumi and Chan [6] found that BIM is the best example of useful innovative technology through which the stakeholders maintain their project information.

**Fig 1:**
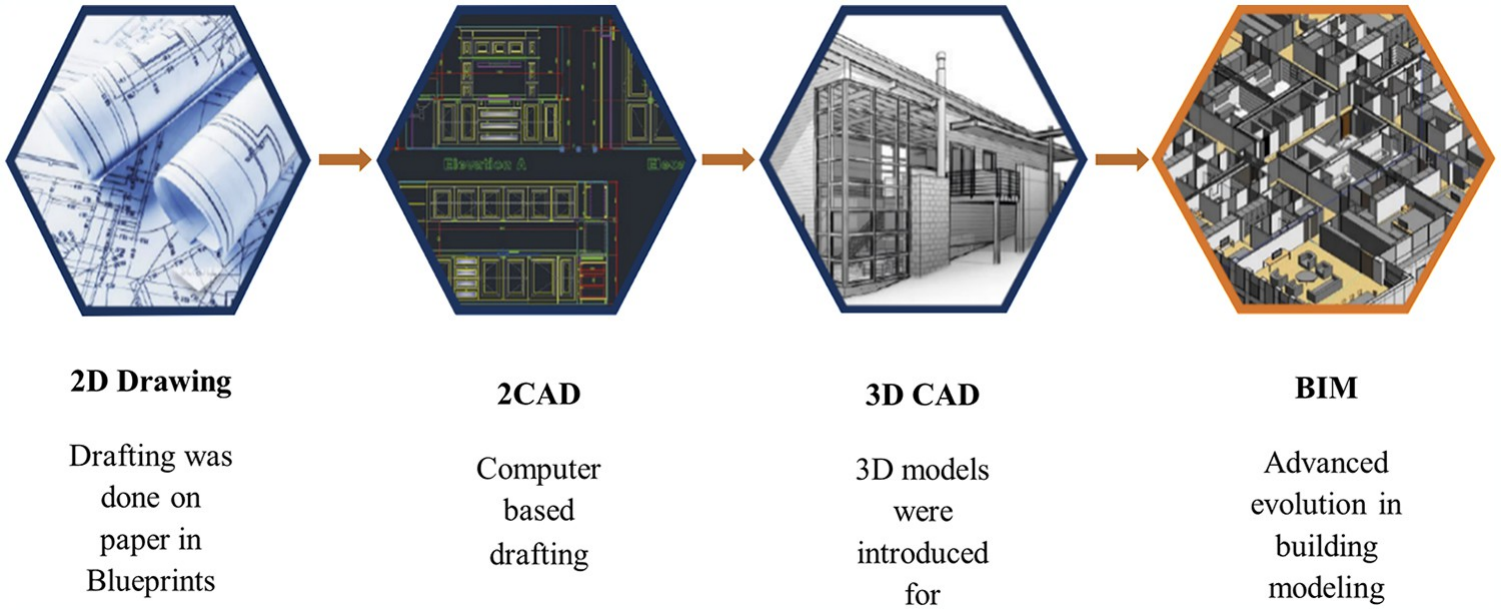
Evolution of BIM with time.

BIM’s evolution from a 3D modeling tool to a comprehensive system including 4D (construction processes and schedules), 5D (cost estimation), 6D (sustainability), and 7D (life-cycle management and maintenance) demonstrate its growing importance in modern construction [1]. Further, the evolution of BIM is shown in Fig 1. BIM’s ability to identify building efficiency, sustainability, and project timelines has proven invaluable, with studies like Hayek [7] defining that BIM can reduce unbudgeted costs by up to 40% in construction projects. The successful application of BIM in developed countries like the UK and the USA has led many construction practitioners to transform from 2D CAD to advanced 7D BIM platforms [8]. This transformation underscores the potential of BIM technologies to improve project outcomes and foster green practices within the construction sector [9]. Since traditional buildings are responsible for 32% of global energy waste and carbon footprints [10], adopting cutting-edge technologies such as BIM can significantly enhance energy performance and mitigate carbon footprints [11].

Despite the numerous advantages of BIM adoption in developed countries, its implementation in emerging countries like the Kingdom of Saudi Arabia (KSA) remains nascent. (KSA), home to one of the largest construction industries, driven by ambitious development projects aligned with "Vision 2030" and supported by a substantial budget exceeding USD 258.8 billion in 2018 [12]. However, a survey by the Ministry of Municipal and Rural Affairs demonstrated that over 70% of construction projects in (KSA) have exceeded their planned timelines, leading to significant delays [13]. Although some studies [14–18] have begun to explore BIM in (KSA), and recognized barriers including organizational, technical, environmental, economic, and social barriers to its implementation [15, 19, 20]. However, a comprehensive analysis of the DFs behind BIM adoption remains lacking. Understanding these DFs is essential for ensuring the successful implementation of BIM technology in (KSA’s) construction projects.

This study addresses this gap by establishing a framework to analyze and investigate the significant DFs that could enhance BIM adoption in the (KSA’s) construction sector. The study’s objectives are to:

- Identifying a comprehensive list of BIM driving forces through an extensive literature review.
- Filter significant driving forces related to BIM adoption using the Fuzzy Delphi Method FDM.
- Analyzing the contextual relationships among various driving forces through the hierarchical structure of ISM.
- Cluster and evaluate the robustness of different DFs using driving and dependence power.

This study makes significant contributions by thoroughly evaluating and prioritizing the DFs that influence BIM adoption in the KSA construction sector. While BIM implementation has been studied in other regions, this research fills a critical gap by focusing on KSA, where BIM adoption is still emerging despite growing demand for modern construction technologies. The novelty of this study lies in its unique application of a combined Fuzzy Delphi Method FDM and Interpretive Structural Modeling ISM approach. This integrated methodology not only screens and prioritizes the key DFs but also maps out the complex interrelationships between them, offering a hierarchical structure that enhances decision-making. By clustering the DFs based on their driving and dependence power, the research provides actionable insights that can guide strategic planning for effective BIM adoption in KSA. Additionally, by contextualizing the findings within KSA’s unique economic, cultural, and regulatory environment, the study contributes to a localized understanding of BIM challenges in current situation. This research also underscores BIM’s role in promoting sustainability, thus aligning with global goals for energy efficiency and carbon footprint reduction, making it particularly relevant in the context of KSA’s Vision 2030.

The structure of this study is as follows. The literature review is discussed in section 2, section 3 is designed to interpret the methodology and results, and the discussion is presented in section 4. Section 5 elaborates on the concluding remarks, theoretical and practical implications, limitations, and future directions.

## 2. Literature review

### 2.1. Driving forces of BIM adoption in construction

It has been seen that the implementation of BIM in construction projects in developing nations is possible through the enforcement of rules and regulations, efficient construction planning and management, and collaboration among all stakeholders in construction projects. However, BIM adoption is at its primitive stage in developing nations, including (KSA). Therefore, BIM can be adopted through proper legislation, increasing the organization’s image, improving quality, and enhancing stakeholder collaboration. Construction practitioners and policymakers can successfully design their planning to achieve BIM adoption in construction projects. The DFs of BIM adoption are discussed below:

#### 2.1.1. Efficient construction planning management

BIM plays a significant role in fostering the efficiency of construction planning and management [10]. The incorporation of BIM enables comprehensive visualization and pre-construction planning, allowing construction practitioners to predict potential issues and optimize resource allocation [8]. Saka, Chan [21] illustrated that BIM mitigates the time and cost related to rework, enhancing the overall productivity of project execution. By incorporating different factors of a project into a single cohesive model, BIM facilitates a smooth construction process, reducing delays and ensuring that projects are finished on time and within budget [3, 22].

#### 2.1.2. Improving safety performance

Safety performance is a significant DF in construction projects, and BIM has been identified for its potential to increase safety precautions significantly [23]. By modeling construction processes, BIM allows the assessment of potential risks before they exhibit on-site [24]. This proactive method enables the advancement of safety measures that eliminate risks related to construction operations [25]. Adopting technology can be beneficial to reduces accidents and incidents on construction projects, contributing to a protected working environment [26]. BIM technology helps the project stakeholders enhance safety performance and safety measures efficiently through which desired goals can be achieved [23].

#### 2.1.3. Increasing organizational image

The implementation of BIM is increasingly identified as a symbol of innovation and technological development in the construction sector [27]. Organizations that adopt BIM are frequently known as industry leaders, which can increase their image and pull new opportunities [28]. Siebelink, Voordijk [29] demonstrated that firms using BIM are better positioned to fulfill the requirements of modern construction, particularly in terms of sustainability and productivity. This positively influences the firm’s reputation, reinforcing its brand and market position [12]. BIM technology pushes traditional organizations to change their operational activities through adopting BIM technology that also helps improve their corporate image in the market [21].

#### 2.1.4. Improving quality

BIM contributes to developing construction quality, offering consistent, precise, and detailed project models that provide a foundation throughout the construction operations [11, 27]. The accuracy of BIM models ensures that the final constriction is affiliated closely with the original design, minimizing the likelihood of inaccuracies and irregularities [9, 30]. Leygonie, Motamedi [31] emphasized that BIM’s capacity to combine different project components into a single model increases the complete quality of construction projects, leading to higher customer satisfaction. Further, Rust, Moorman [32] claimed in broader literature improving quality can lead to cost reduction through enhanced efficiency and reduced rework.

#### 2.1.5. Reducing costs

One key DF of BIM implementation is its potential to minimize construction costs [7]. BIM enables accurate cost prediction and budgeting, allowing better financial planning and resource management [2, 33]. By minimizing the need for rework and reducing material waste, it also assists construction organizations in managing financial control over their projects [28, 34, 35]. Saka and Chan [4] demonstrated that consistency encourages BIM’s cost-saving benefits and fosters the economic viability of construction projects.

#### 2.1.6. Visualization of data

BIM’s visualization capabilities are essential for productive project management and decision-making [36]. By delivering transparent 3D representations of construction projects, BIM allows stakeholders to understand the project’s scope and progress [24]. This increased visualization fosters communication and collaboration among project teams, mitigating the likelihood of misunderstanding and errors [37]. Ariono, Wasesa [38] analyzed that BIM visualization tools are valuable in keeping projects aligned with design specifications and timelines. According to Yang and Zhao [21], the potential drivers of BIM can be increased through utility, speed, data visualization, and enhanced fault finding.

#### 2.1.7. Enhancing collaboration among stakeholders

Coordination among stakeholders is essential DF for the success of construction projects. BIM supports this by offering a cooperative platform where all stakeholders can access and share information in real time [39]. This clarity and ease of communication assist in aligning stakeholders with the project goals, mitigating the risk of conflicts and delays [40, 41]. Aziz and Zainon [8] highlighted the significance of BIM in improving productive collaboration, which leads to more meaningful and successful project results.

#### 2.1.8. Increasing sustainability

Sustainability is a growing issue in the construction sector, and BIM is vital in promoting green practices [42]. BIM enables the energy utilization and material efficiency model, allowing construction projects to reduce their environmental influence. Shojaei, Oti-Sarpong [43] indicated that BIM contributes to designing and constructing eco-friendlier buildings, aligning with international struggles to minimize carbon footprints and protect resources. Sustainability-related DFs of BIM can be enlisted as wastage reduction by the optimum level of inventory and cost [2]

#### 2.1.9. Controlling whole-life costs and environmental data

BIM’s capacity to maintain and handle whole-life costs and environmental data drives construction projects’ long-term progress [43]. By following the life cycle of a building from design through to operation and management, BIM allows more precise cost estimation and environmental influence evaluation [44]. Zakeri, Tabatabaee [1] analyzed that BIM is productive in mitigating unpredicted costs and ensuring that environmental objectives are fulfilled throughout the project life-cycle.

#### 2.1.10. Reducing labor work

BIM’s automation capacities significantly minimize manual labor requirements in construction projects [11]. By automating tasks such as quantity takeoffs, cost forecasting, and scheduling, BIM enables employees to concentrate on more intricate and value-added activities [4]. Goel [2] demonstrated that reducing manual labor leads to enhanced efficiency and improvement on construction sites. BIM technology minimizes labor involvement in construction projects that cause delays in building projects [4].

#### 2.1.11. Optimizing the owner’s experience and satisfaction

BIM increases the owner’s experience by offering a transparent and interactive platform where they can handle project performance and contribute to decision-making [45]. This type of participation ensures that the project fulfills the owner’s expectations, leading to higher satisfaction levels [43]. AbuMoeilak, AlQuraidi [46] emphasize that BIM’s capacity to facilitate communication and collaboration between owners and project managers is a key DF to accomplishing successful project results. Saka and Chan [21] explain that BIM implementation builds trust among contractors and owners of projects and helps optimize their experience and satisfaction level.

#### 2.1.12. Reducing document errors and omissions

Document errors and omissions are common sources of delays and cost overruns in construction projects [47]. BIM advocates this DF by centralizing all project information within individual models, ensuring all stakeholders can access precise and fresh data [12]. Egwim, Alaka [48] identified the efficiency of BIM in minimizing document-related issues, leading to straightforward project completion. Cao, Li [27] assumed that a human-related error could be minimized by adopting BIM technology in construction projects.

#### 2.1.13. Reducing the cycle time of the design process

BIM significantly lessens the design procedure by enabling real-time cooperation and allowing for incorporating different design factors within a single model [43]. This mitigates the need for revisions and leverages the comprehensive project timeline [48]. Hong, Hammad [49] analyzed that BIM’s influence on mitigating design cycle time is especially advantageous in intricate projects where collaboration is significant. BIM technology extends the prospect of developing better cost estimations based on essential components of the construction process, improved design, construction procedures, and approaches, and is a means to engage the client in the design segment of the construction process [4]. Chang, Kang [24] observed that effective BIM implementation yields crucial insights for enhancing design, optimizing design processes, facilitating integrated project delivery, and enhancing building performance.

#### 2.1.14. Better understanding of energy consumption

BIM delivers a robust tool for examining and enhancing building energy consumption [11]. By modeling various energy utilization perspectives, BIM assists stakeholders in making informed decisions that increase energy efficiency and decrease operational costs [22]. Zakeri, Tabatabaee [1] underscored the role of BIM in accomplishing green energy management in construction projects. Olawumi and Chan [6] reported that BIM could enhance environment-friendly activities in construction projects by summarizing energy consumption in buildings. Saka and Chan [4] elaborate that a better understanding of energy patterns during construction projects helps maximize projects’ success.

#### 2.1.15. Reducing risk

Risk management is the critical scenario of construction projects, and BIM has been presented to substantially minimize different risks related to designs, costs, and scheduling [36]. By delivering an overall model of the project, BIM facilitates the earlier identification and elimination of potential risks, contributing to the comprehensive accomplishment of the project [34]. Ali, Hegazi [39] defined the utilization of BIM as a productive tool for risk elimination in construction. Karacigan, Ozorhon [50] developed a cross-sectional questionnaire to examine the advantages of 5D BIM. The findings from this questionnaire highlighted improvements in efficiency, visualization, and early risk identification within the field of quantity surveying [27].

The literature on BIM implementation discloses the broader adoption of DFs that substantially impacts its uptake in the construction sector. These DFs, from technological innovation to regulatory framework, play a significant role in increasing project efficiency, fostering safety, and promising ecology. However, the influence and prominence of these DFs fluctuate across various areas, especially between developed and developing nations.

In developed nations such as the US, the UK, and Germany, BIM implementation is strongly fueled by innovative technological infrastructure, robust regulatory mechanisms, and a well-designed prominence on sustainability [51–53]. Government policies are a significant driver, with strategies needed for BIM utilization in public construction projects leveraging adoption. For example, the UK’s policies for BIM level II in all public projects have enhanced BIM utilization and drew a benchmark for other nations to follow [54]. Further, these countries benefit from high levels of technological advancement, which includes easy access to advanced software and hardware and skilled experts who can utilize BIM’s full potential [55]. Economic subsidies, such as tax exemptions and incentives, further encourage the transformation to BIM, making it an attractive proposition for construction industries. The concentration on sustainability is another major DF, with BIM seen as significant for accomplishing energy efficiency and mitigating carbon footprint, which aligns with the broader environmental goals of these countries [56].

On the other hand, the situation in developing nations, such as Sri Lanka, Bangladesh, and India, presents a different set of issues and priorities [57–59]. In these countries, the implementation of BIM is primarily motivated by the need to minimize construction costs and foster project efficiency. Given the often limited resources and infrastructure, the ability to provide projects on time and within budget is a significant issue [60]. Government initiatives to foster BIM are gradually developing, but they lack the uniformity and enforceability seen in developed nations [61]. Further, the technological infrastructure in many developing countries is not innovative and modern, hindering BIM adoption [62]. Therefore, training and capacity-building initiatives are significant, as these nations often lack skilled experts who can effectively adopt and maintain BIM technologies [63]. Another key DF in these countries is the desire to increase international competitiveness. As these countries aim to align with global standards, implementing BIM becomes a strategic move to attract foreign investment and collaborate more actively in international construction markets [64].

The extensive review of the current literature on BIM implementation in the construction industry within emerging nations discloses different major DFs that are consistently known as significant for successful adoption. Among these DFs, efficient construction planning and management are frequently emphasized. BIM’s ability to optimize project timelines, increase resource allocation, and reduce delays is prevailing across multiple studies. Another crucial DF is the reduction in labor work, as BIM automates different requirements and minimizes the risk of human error. Safety performance improvement is also extensively discussed, with various studies highlighting how BIM’s detailed visualization and simulations contribute to proactive safety measures.

Furthermore, cost minimization is recognized as an essential DF for BIM implementation, with its ability to reduce waste, increase material use, and prevent costly rework being well documented. The literature also highlights the significance of BIM in delivering a better awareness of energy consumption, which enables the design of energy-efficient buildings and encourages green construction projects. Additionally, BIM’s increased data visualization capabilities, including 3D models to 7D, are necessary for enhancing project communication and stakeholder collaboration, leading to more informed decision-making throughout the project life-cycle. Finally, increased sustainability is underscored as a major DF, minimizing carbon footprint and fostering the overall performance of construction projects. These multifaceted DFs underscore the crucial role of BIM in driving advancement and efficiency in the construction industry. Table 1 shows the DFs from earlier studies.

**Table 1:** Drivers of BIM adoption and their frequency.

No.	BIM Drivers	Description	Frequency	References
DF1	Efficient construction planning and management	Increases project coordination, scheduling, and resource management to foster comprehensive efficiency	Moderate	[3, 10, 21, 22]
DF2	Improve safety performance	Increases safety by modeling construction processes and analyzing possible risks.	High	[1, 23–25, 42]
DF3	Increase organization image	Foster the image and competitiveness of the firm by adopting cutting-edge technologies.	Moderate	[21, 27, 29, 65]
DF4	Improve quality	Improve the quality of construction projects through accurate and reliable design and implementation.	High	[9, 11, 27, 30, 31, 66]
DF5	Reduce cost	Reduce comprehensive project costs by increasing resource utilization and minimizing waste.	High	[2, 4, 7, 28, 34]
DF6	Visualization of data	provide 3D modeling and visualization tools for better decision-making and project management.	Moderate	[24, 36, 37, 50]
DF7	Enhance collaboration among stakeholders	Enables better communication and cooperation between various project stakeholders.	Moderate	[8, 7, 39, 40]
DF8	Increase sustainability,	Endorses green construction practices by enhancing resource utilization and mitigating carbon footprint.	High	[3, 9, 25, 36, 42, 67]
DF9	Controlled whole-life costs and environmental data	Ensures that projects are economically and environmentally sustainable over their entire life-cycle.	Low	[1, 43, 44]
DF10	Reduction in labor work	decreases manual labor through automation and digital tools, leading to cost and time savings.	Low	[2, 4, 28]
DF11	Optimize the Owner’s experience and satisfaction	Increases customer satisfaction by providing projects on time, within budget, and to an excellent quality.	Moderate	[43, 45, 46, 68]
DF12	Reduction in documents error and omission	Reduces errors and omissions in project documentation through digital processes.	High	[27, 28, 45, 47]
DF13	Reduced cycle time of the design process	Boost the design process by allowing more productive workflows and minimizing delays.	Low	[48, 49, 69]
DF14	Better understanding of energy consumption	Delivers insights into energy utilization, facilitating the design of more energy-efficient buildings.	High	[1, 4, 6, 11, 22]
DF15	Reduce Risk	Assists to analyze and handle risks throughout the project life-cycle, minimizing potential risks.	High	[27, 28, 34, 36, 39]

### 2.2. Research gap

Although the implementation of BIM has already been investigated in some developing countries, studies were underrated because of the following issues. For instance, there is a lack of using novel methodologies, a lack of comparative analysis, and a limited focus on organizational factors. Overall, the literature shows different aspects of BIM implementation, such as various benefits, driving forces, and strategies [70]. However, these studies lack consistency in defining and categorizing the driving forces of BIM [49]. Different studies have used a variety of frameworks, which has led to fragmented and inconsistent results. These results are difficult to compare and synthesize due to the absence of standardization. Research on the deployment of BIM has concentrated exclusively on identifying the drivers or hurdles without thoroughly examining how they interact [71]. This focused approach constrains studying the intricate dynamics and interactions between these DFs. The application of BIM necessitates cross-disciplinary cooperation encompassing organizational, managerial, and technological elements. However, the material that is now available frequently concentrates on technical drivers while ignoring the more significant organizational and management issues that affect the adoption of BIM [5]. Interdisciplinary viewpoints should be incorporated into a thorough analysis to fully represent the complex interaction of driving forces across various dimensions [44].

## 3. Methodology

**Fig 2:**
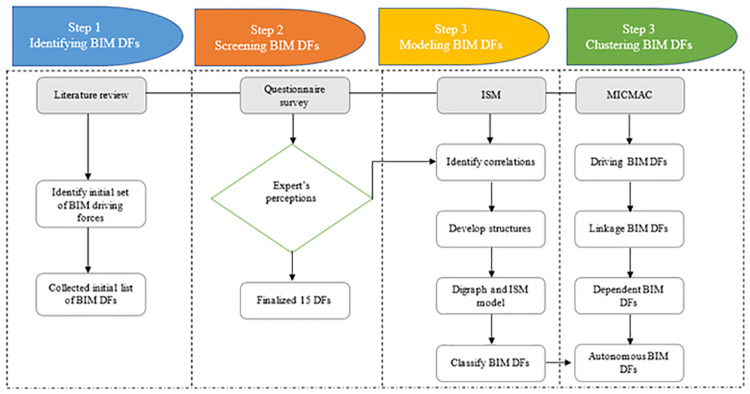
Research framework.

**Fig 3:**
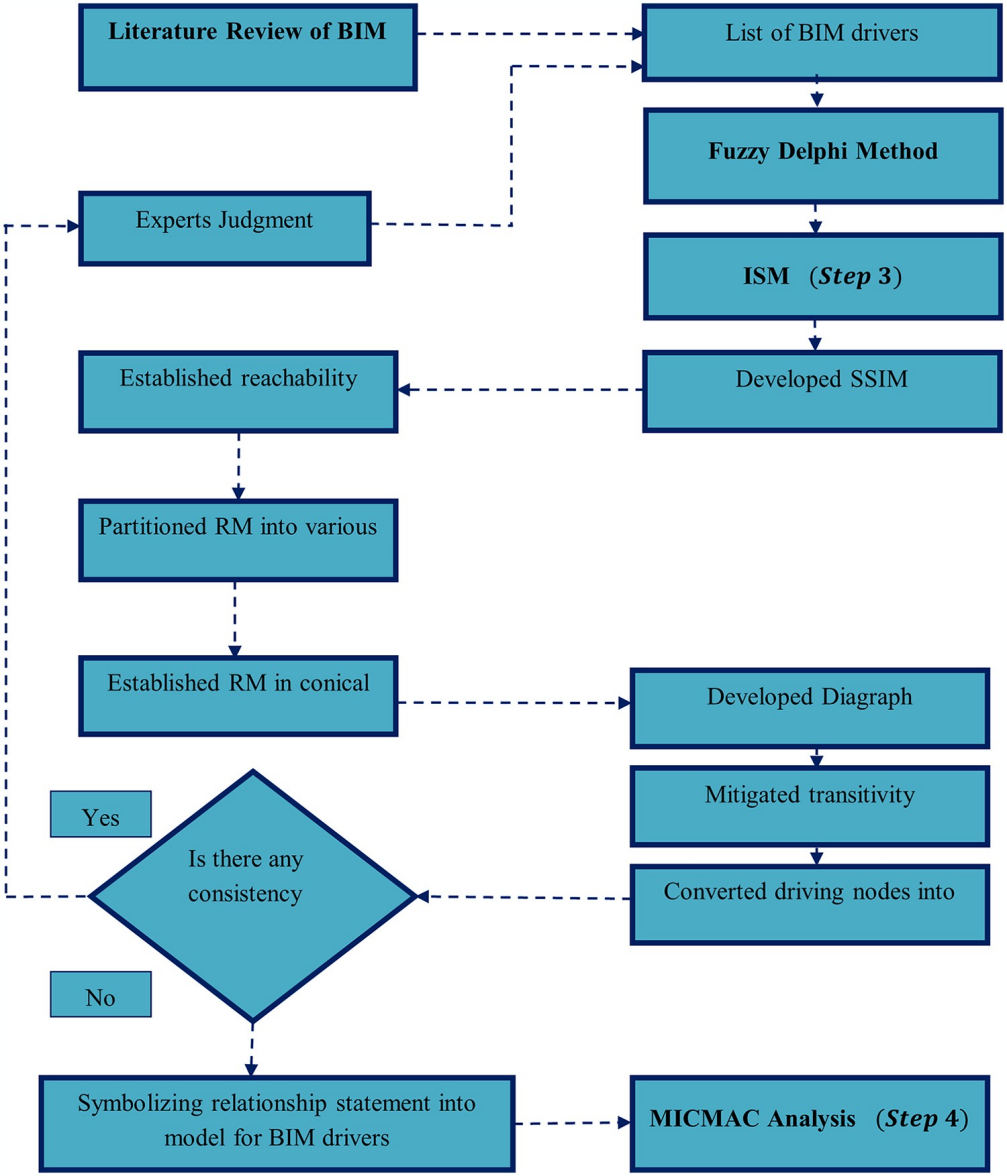
Step by step methodology.

This study adopted four research methods: literature review, FDM, ISM, and MICMAC approach. The flow diagram of these integrated methodologies has been given in Fig 2. The literature review explores the significant leading elements from previous research studies. FDM and ISM-MICMAC are integrated in this study, and the FDM was used to screen the DFs identified in earlier studies. ISM was utilized to examine the complex relationship of different DFs and their influence on each other. Further, MICMAC analysis was finally followed to investigate the dependence, driving capability, and influence. The step-by-step methodology of the study is presented in Fig 3.

### 3.1. Exploring DFs from earlier literature

The literature review follows a systematic, transparent, and reproducible approach to classifying, assessing, investigating, and interpreting previous work, as recommended by leading guidelines [65]. The purpose of this study was to identify the possible major DFs of BIM. To achieve this; databases such as Google Scholar, Science Direct, the Web of Science, Scopus, Emerald, and Springer were used. To recover all relevant data in the database, the author used various terms such as BIM benefits, BIM advantages, and BIM drivers, through which 980 articles were explored. All these articles were scrutinized to assess whether they meet the following requirements: (1) papers should be published in reputed journals, (2) the topic of the research articles should belong to BIM benefits, BIM drivers, and BIM issues. Finally, 68 out of 980 articles were selected for further study. The search criterion is given in Table A1 (S1 Dataset). The frequency listed 15 DFs of BIM adoption in different contexts, are already shown in above Table 1. The "frequency" column in Table 1 showed the significant existence of each (DF) in the reviewed literature. This was examined by counting the number of scholarly articles and studies that focused on each DF in the context of BIM implementation. For example, a DF categorized as "High" was frequently emphasized through a substantial number of sources, while "Moderate" or "Low" indicates less frequent but still relevant mentions in the literature. After exploring significant DFs from the previous studies, the next step is to filter the most pertinent DFs. For this purpose, FDM was used, which will be discussed in the next section.

### 3.2. Filtering factors through the Fuzzy Delphi Method (FDM)

Delphi Method DM has been employed in different domains of the research. While the DM leverages experts’ valuable insights, it possesses certain limitations. Notably, it is known for consuming a substantial amount of time, potentially impeding decision-making [72]. Moreover, there is a possibility of limited convergence in the DM, it can incur high execution costs, and it relies on experts providing comments during discussions, introducing the potential for divergent opinions [72].

According to Murray, Pipino [73], Fuzzy theory helps reduce the ambiguity of the DM, and Delphi was combined with fuzzy, known as the fuzzy Delphi method (FDM). Hsu and Yang [74] have noted that incorporating fuzzy numbers can enhance the effectiveness of decision-making processes within this context. The FDM approach has been widely used in business-related studies because experts’ opinions endorse decision-making on factors such as barriers, strategies, and drivers [75].

FDM permits experts to fine-tune their responses, thereby minimizing the array of factors that exert influence and enabling the identification of the most pertinent factors [76]. Further, this approach assists in getting a consensus among respondents. In this study, FDM filtered the significant DFs regarding the adoption of BIM technology. Application of FDM for DFs supporting BIM in the construction sector is given below:

#### 3.2.1. Designing and piloting the questionnaire

The questionnaire for the study was formulated using the derived DFs obtained from an extensive review of the existing literature. Subsequently, these DFs were compiled and submitted to 6 academic experts to evaluate their content validity in round one. The appropriateness and most relevancy of DFs were checked in the second round. Further, four out of six academicians later participated as members of the experts’ panel in the subsequent phases of the study. Their primary role involved scrutinizing the questionnaire for any discrepancies or inconsistencies. Following their validation and approval, the questionnaire was deemed suitable for distribution among the targeted group of experts.

#### 3.2.2. Questionnaire distribution

**Fig 4:**
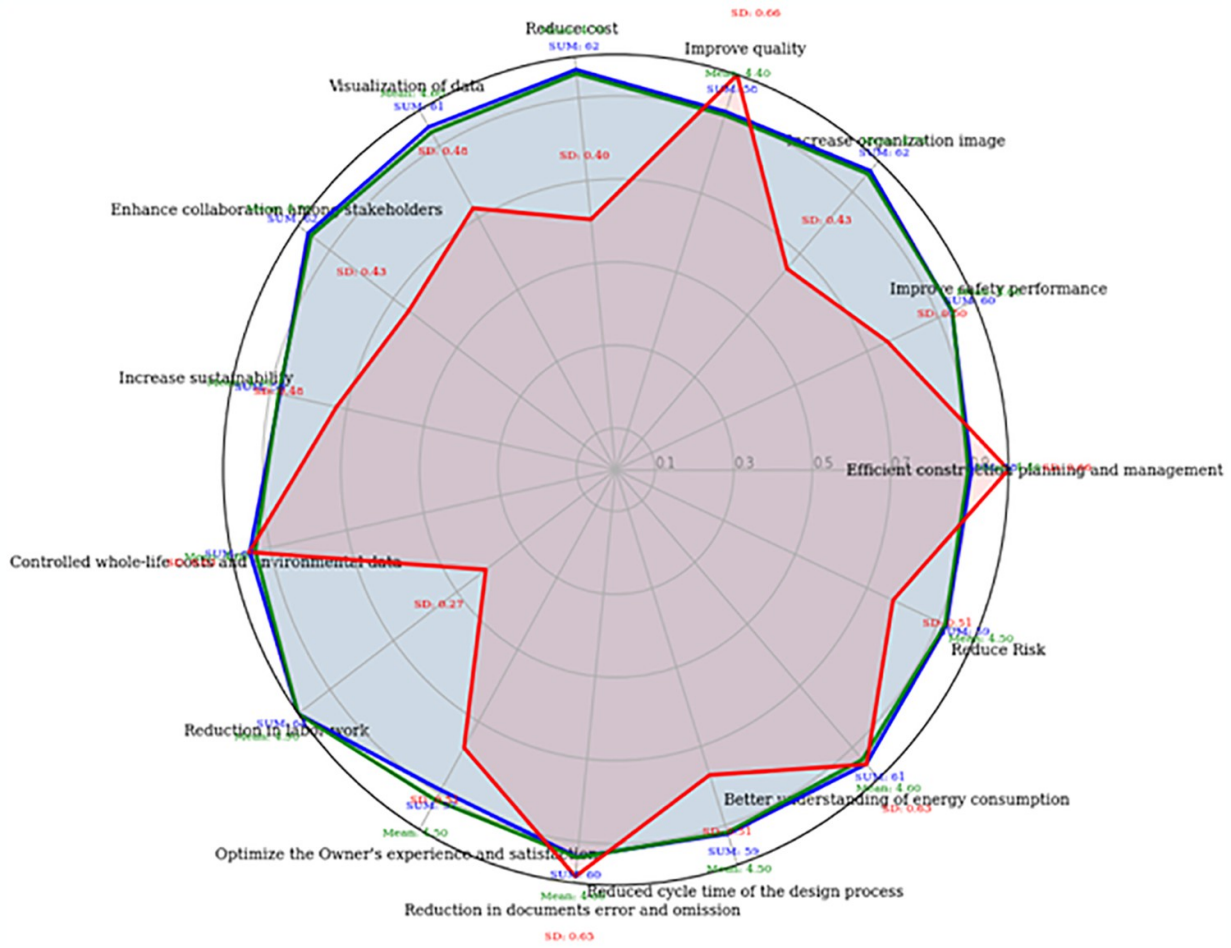
Filtering suitable DFs through FDM’s experts ranking.

The questionnaires were distributed between experts from the KSA construction sector and academicians from different institutes of KSA. In this procedure, a threshold of 52 was chosen as the score cutoff point, as this criterion was also employed by [76]. The DF that Scores were equal to or greater than 52 were deemed acceptable (as shown in Fig 4). All the DFs’ scores were more than 52, so all 15 DFs were retained to continue for further study. During this procedure, the expert’s suggestions regarding the adjustment, removal, and inclusion of DFs were also considered.

The results of FDM are presented in Table A2 (S1 Dataset). Furthermore, a five-point Likert scale was implemented to mitigate potential bias during the data collection and ensure the data’s validity.

#### 3.2.3. Managing expert’s opinions and developing the Triangular Fuzzy Numbers TFNs

This study adopted the TFNs calculation method as outlined in [75]. TFNs were employed to determine experts’ opinions’ maximum and minimum values. Geometric means were employed to arrange the TFN results sequentially to ensure clarity. The geometric mean involves listing experts’ assessments’ minimum and maximum values. In this study, the GM represents the professional’s judgment.

Suppose $W_{j}^{n}=(a_{j}^{n}\;b_{j}^{n}\;c_{j}^{n})$ exemplify the preference of each expert BIM DFs *j* developed by “*nth*” assessment of experts in TFNs. To integrate the choice of all “*n*” assessment, the Eq (1) will be utilized as

$$W_{j}=\left(a_{j}b_{j}c_{j}\right)=\left(a=\text{min}\left\{a_{ij}\right\},b_{j}=\;\frac{1}{n}\;\sum_{i=1}^{n}b_{ij},c_{j}=\text{max}\left\{c_{ij}\right\}\right)$$ (1)

As *w*_*j*_ Represent cumulative TFNs.

#### 3.2.4. Selection of drivers

The final section provides TFNs for each driver, the GM, and the minimum and maximum values of experts’ judgment. The thumb rule was employed to explore the significant DFs [75]. In this process utilizing the FDM, the essential drivers are recognized by comparing the weight of each driver with the threshold. ~_*w*_. ~_*w*_ present the average weight of DFs regarding BIM adoption.


$$w_{j}=\frac{a_{j}+b_{j}+c_{j}\;}{a},j=1,2,3,\ldots m$$ (2)

As "w" is denoted as a crisp score representing the aggregate preference of each expert BIM driver j.

Suppose *a*_*j*_ ≥ *w*, then the DFs *j* is accepted.

Suppose *a*_*j*_ < *w*, then DFs *j* is excluded. After collecting data from experts, the main drivers were filtered. Therefore, all the DFs were retained for further process after applying FDM.

### 3.3. Interpretive Structural Modeling (ISM)

ISM is primarily utilized to illustrate the interconnections among variables, especially in cases where issues are intricate or multifaceted [77]. Nevertheless, it’s worth noting that several Multi Criteria Decision-Making (MCDM) techniques can also conduct similar analyses [78], as shown in Table A3 (S1 Dataset). The preference for ISM over other MCDM approaches in this study can be attributed to several factors. ISM is particularly effective in dealing with intricate interrelationships among variables and provides visual representations that enhance comprehension [79]. Additionally, ISM can incorporate qualitative insights, facilitate the identification of hierarchical relationships among DFs, and offer simplicity in its implementation [80]. In certain instances, ISM can be a more straightforward choice for implementation and interpretation compared to other MCDM methods, which often demand extensive quantitative data and involve complex mathematical computations [81]. ISM helps prioritize elements by classifying the most influential ones, which can significantly impact decision-making processes [82]. Furthermore, the ISM-MICMAC approach has been widely adopted in various domains. For example, Iqbal, Waqas [83] employed this approach to identify the challenges of sustainable supply chain operations in the manufacturing industry. Similarly,Iqbal, Ma [84] utilized the ISM-MICMAC technique to establish policies for relaxing lockdown restrictions in response to COVID-19. Alaboud and Alshahrani [85] applied the ISM approach in the construction industry to investigate BIM implementation factors. Naheed, Waqas [86] employed ISM to reveal the significant barriers that hinder the adoption of sustainability and green innovation in the manufacturing industry. Moreover, Hussain, Sun [87] used ISM-MICMAC to investigate the risk factors of green lean six sigma from the perspective of the construction industry. These examples illustrate the robustness of ISM-MICMAC in structuring and analyzing complex, interrelated issues, reinforcing its suitability for this study, which seeks to explore and prioritize the driving factors of BIM implementation in the construction sector. By referencing these prior applications, this research builds on a well-established methodological foundation. Further, the application of ISM is included in different steps, which are given below:

#### 3.3.1. Formation of structural self-interaction matrix (SSIM)

SSIM is a pairwise relationship among the elements. It was developed with the assistance of experts’ opinions through a questionnaire; a questionnaire was developed and given to experts to get their opinions regarding the contextual relationship of different DFs with each other. The data was collected from experts in the construction industry of (KSA). Initially, we continuously contacted 30 experts through various modes such as LinkedIn, official emails, Phone calls, and Twitter. We often communicated with the experts to respond to questions through phone calls and wrote many emails as reminders. At least 13 out of 30 experts consented to participate in data collection through Zoom meetings. A group of experts included three consultants, two civil engineers, four architects, three professors, and one associate professor. These selected experts were awarded the highest degree, having sound experience in the subjective field and decision-making ability. The list of experts is shown in table A4 (S1 Dataset).

**Fig 5:**
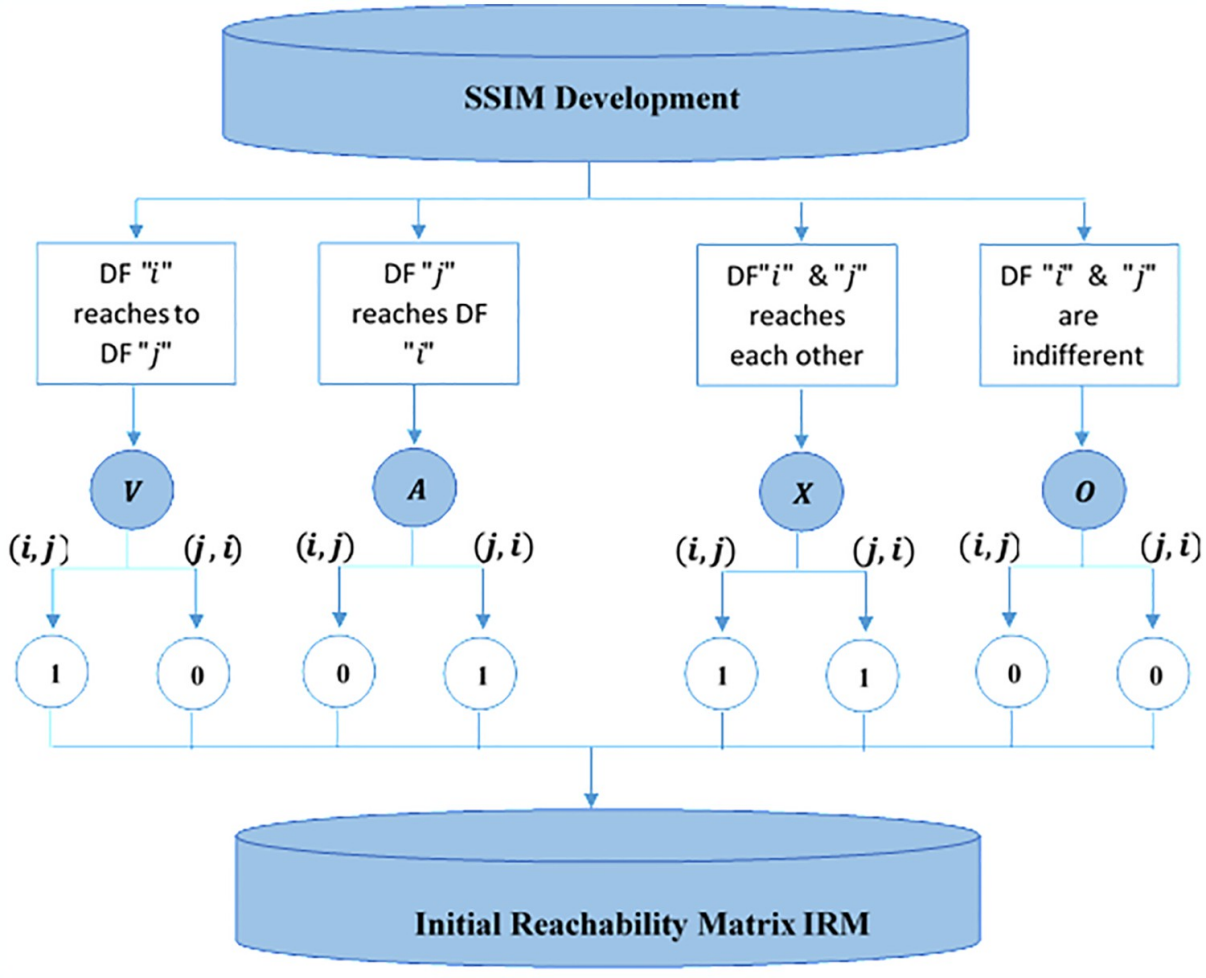
Establishment of SSIM into IRM and FRM.

*VAXO* format was adopted to present directional relations between two DFs. The four symbols were used in *VAXO* scale as *V* represents that ’*i*’ reaches factor ’*j*,’ *A*: is used to show that ’*j*’ reaches factor ’*i*.’ *X* expresses that both ’*i*’ and ’*j*’ get each other, and *O* directs that both ’*i*’ and ’*j*’ have no reach each other. Professional feedback on SSIM is presented in Table 2. The basic concept of establishing SSIM after the conversion of SSIM into IRM and IRM into FRM is presented in Fig 5.

**Table 2:** Self-structural interaction matrix SSIM.

SR. NO	Drivers	1	2	3	4	5	6	7	8	9	10	11	12	13	14	15
**DF1**	Efficient construction planning and management		V	V	V	V	X	V	V	V	X	V	V	V	V	V
**DF 2**	Improve safety performance			V	X	V	X	A	V	A	V	V	O	O	X	V
**DF 3**	Increase organization image				A	A	O	A	A	A	O	A	A	A	V	X
**DF 4**	Improve quality					O	A	A	V	V	A	X	V	V	V	A
**DF 5**	Reduce cost						A	A	O	A	A	A	A	A	O	X
**DF 6**	Visualization of data							V	O	X	O	V	V	V	V	V
**DF 7**	Enhance collaboration among stakeholders								X	V	V	X	V	V	V	V
**DF 8**	Increase sustainability									A	A	V	A	A	V	V
**DF 9**	Controlled whole-life costs and environmental data										A	V	O	A	A	O
**DF 10**	Reduction in labor work											O	O	V	O	V
**DF 11**	Optimize the Owner’s experience and satisfaction												A	A	A	A
**DF 12**	Reduction in documents error and omission													A	A	V
**DF 13**	Reduced cycle time of the design process														O	O
**DF 14**	A better understanding of energy consumption															V
**DF 15**	Reduce Risk															

#### 3.3.2. Initial reachability matrix (IRM)

The initial reachability matrix is designed through the entries of binary digits(0,1); the transformation of alphabets into binary digits is based on certain logic, which is defined as

1. If *V* exists in SSIM, the binary digit 1 will be entered in (*i*, *j*), and 0 will be entered in(*j*, *i*).
2. If *A* exists in SSIM, it means the binary digit 0 will be entered in (*i*, *j*), and 1 will be entered in(*j*, *i*).
3. If *X* exists in SSIM, it means the binary digit 1 will be entered in both entries(*i*, *j*), and (*j*, *i*).
4. If *O* exists in SSIM, it means the binary digit 0 will be entered in both entries(*i*, *j*) and (*j*, *i*).

According to this process, IRM table A5 (S1 Dataset) is formulated.

#### 3.3.3. Final Reachability Matrix (FRM)

After the completion of the IRM, an additionally incorporated transitivity rule is needed to establish the FRM with the entry of 1* Table A6 (S1 Dataset) shows that FRM supports obtaining driving and dependence power by calculating the sum of column and row entries. Both powers support the development of the ISM level-based model and factor clustering, which is analyzed through the MICMAC approach.

#### 3.3.4. Level partition

The final reachability was continued to partition the factors into numerous levels and determine the hierarchy of variables. Both the antecedent and reachability sets are taken from the FRM of each factor. The reachability set combines itself and other factors that may help reach it; the antecedent set contains different elements that may support achieving it. The intersection set is established through the integration of reachability and antecedent sets. Suppose the given factors are the same in reachability and intersection set. In that case, the level is assigned to those factors, which is removed to continue the further iteration procedure. This iteration procedure is continued until all factors are achieved. The level iteration of all DFs is presented in Table A7 (S1 Dataset). From the outcomes of level partition, hierarchical levels were obtained, as shown in Table A8 (S1 Dataset).

#### 3.3.5. Formulation of finalized digraph model

**Fig 6:**
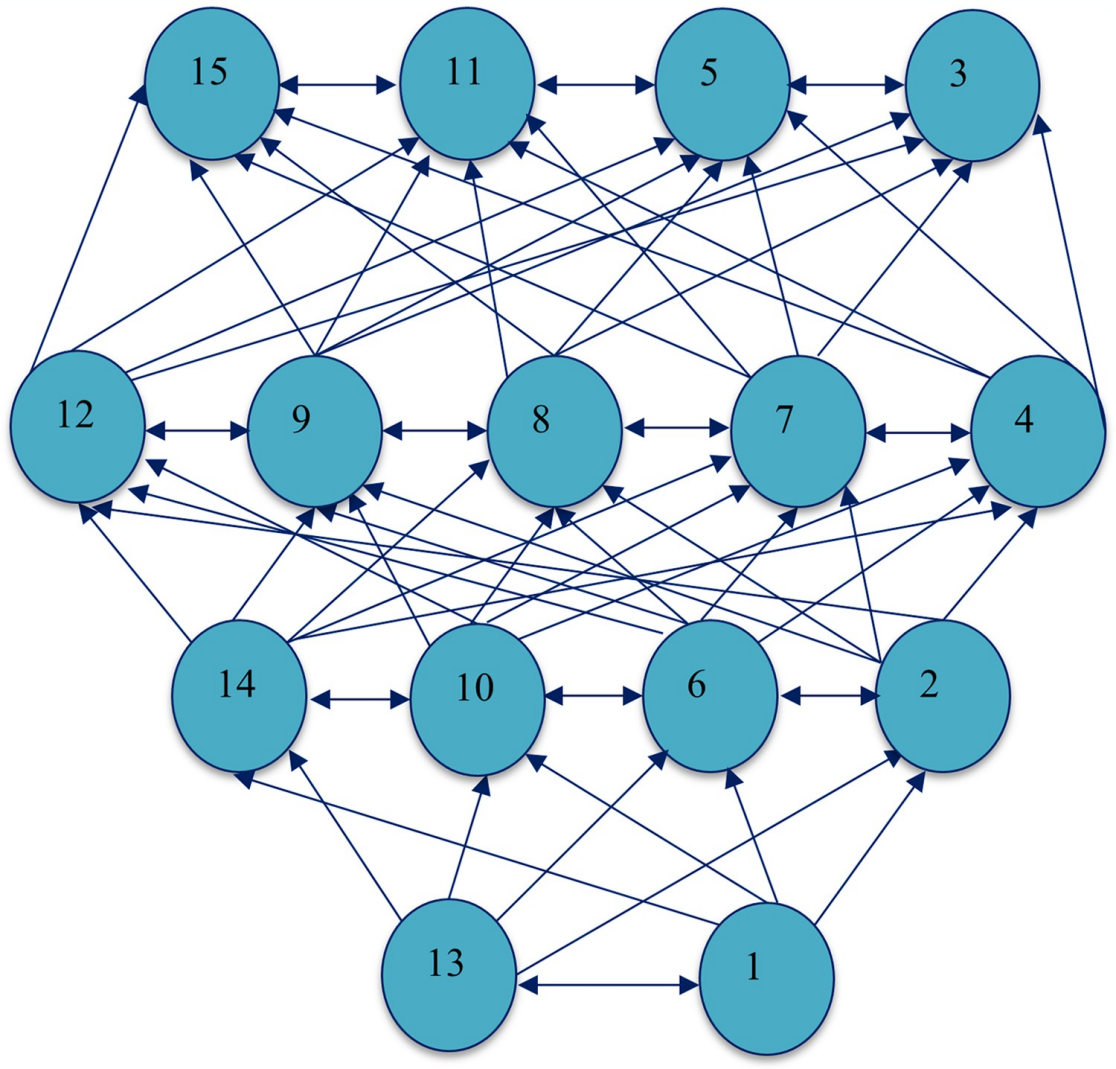
Depicted diagraph of BIM DFs.

**Fig 7:**
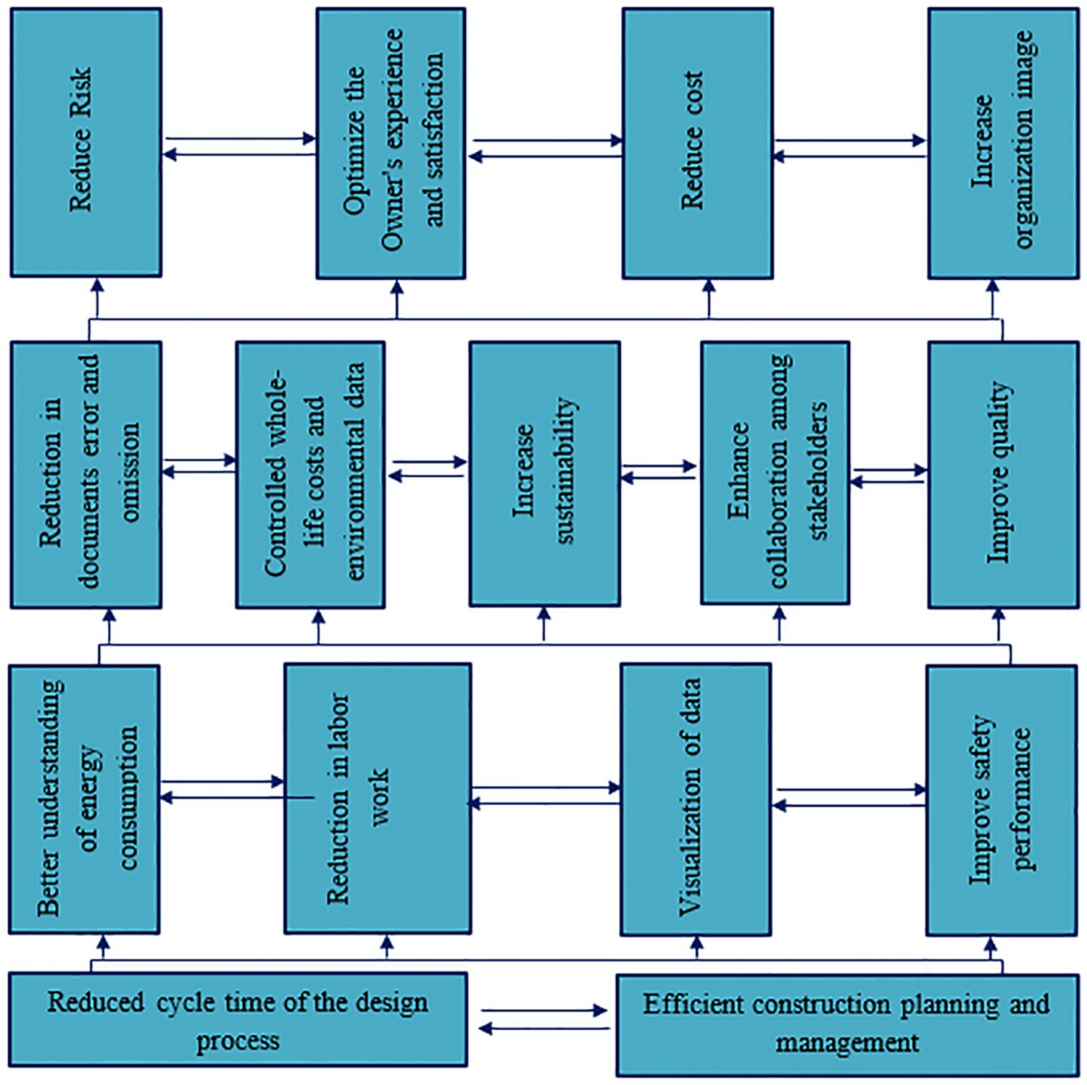
ISM-based model of DFs to BIM adoption.

After extracting the level partition, the last step was formulating the final ISM model. Transitivity was removed from the initially depicted digraph, presented in Fig 6, and that digraph was changed into the Final ISM hierarchical structural framework, as presented in Fig 7.

### 3.4. MICMAC analysis

**Fig 8:**
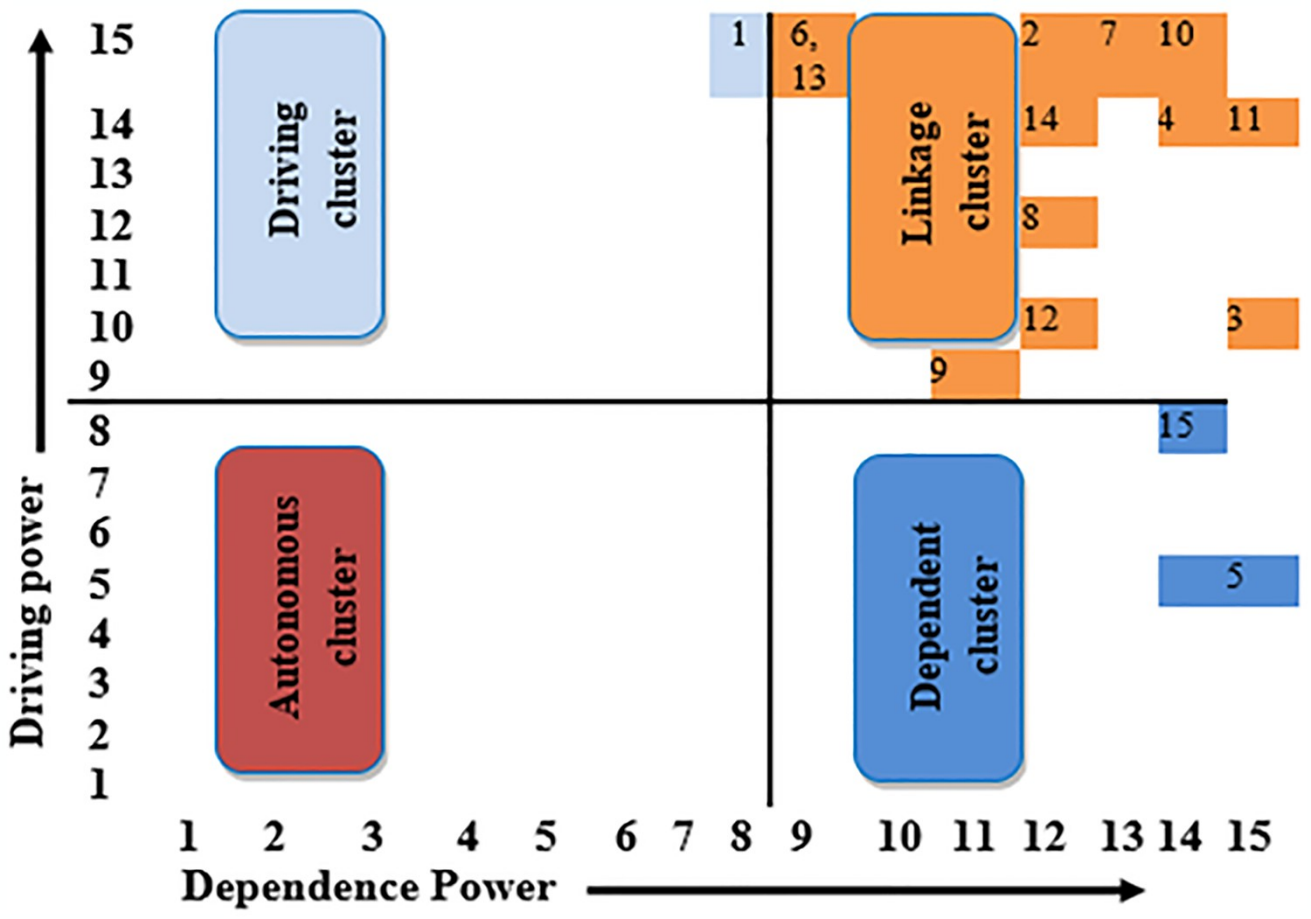
MICMAC analysis of BIM DFs in construction projects of KSA.

**Fig 9:**
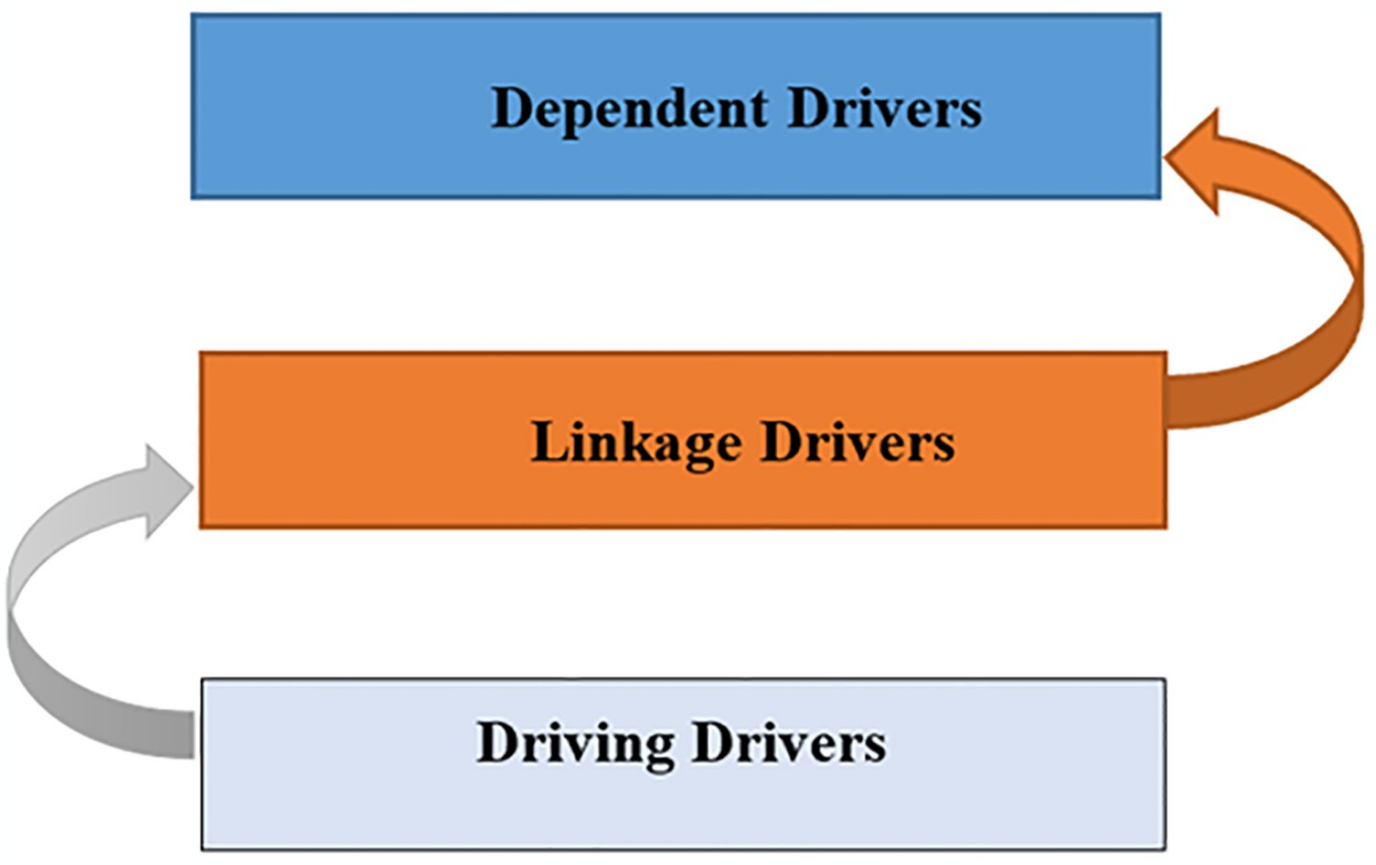
Relationship between the segments of drivers.

MICMAC analysis aims to analyze the factors affecting dependence and driving power. The final Reachability matrix is the primary source to determine the dependency and driving powers by putting binary numbers (0,1) in rows and columns and their interpretation and calculation. According to dependence and driving power, MICMAC was executed in four categories: driving, linkage, autonomous, and dependent, as shown in Fig 8. To comprehend the interactions of MICMAC clusters, see Fig 9. By classifying these DFs into four clusters, the MICMAC analysis assists in:

- It enables identifying drivers that can be targeted for interventions to impact the entire system effectively.
- The analysis highlights how various drivers interact, which is critical for establishing policies responsive to the system’s intricate nature.
- By categorizing which DF has the significant influence and which relies on others, the analysis aids in prioritizing actions and resource allocation to accomplish desired results more efficiently.

#### 3.4.1. Driving cluster

This cluster of factors has weak dependence power and strong driving power. In this study, one driver known as efficient construction planning and management DF1 was classified as the driving driver, demonstrating their primary role in shaping the other DFs of the systems.

#### 3.4.2. Linkage cluster

Linkage DFs were characterized by high impact and dependence within the system. This dual nature means that other drivers primarily influence these DF and significantly affect them. Consequently, they tend to be highly sensitive and unstable, often creating feedback loops within the system. In this study, twelve DFs, including DF6, DF13, DF2, DF7, DF10, DF14, DF4, DF11, DF8, DF12, DF3, and DF 9, were categorized as driving drivers, representing their primary role in shaping the other drivers of the system. For instance, the DF 6 can have cascading effects on other drivers but can also be influenced by changes elsewhere in the system.

#### 3.4.3. Autonomous cluster

This cluster has both weak powers as dependence and driving power, illustrating that they are substantially disconnected from the system. In this study, no driver was found to be autonomous, which recommends that all the examined drivers be interconnected and play a significant role in the system.

#### 3.4.4. Dependent cluster

This cluster of elements has a robust dependence force and weak driving force and is positioned at the top of the ISM hierarchical model. The drivers that exist in dependent clusters often are influenced by other drivers. For instance, DF15 and DF5 fall into dependent clusters, demonstrating that while they are significant to the system, they depend heavily on other DFs to occur.

## 4. Results and discussion

BIM technology’s adoption in construction projects has grabbed the attention of different stakeholders in the last few decades. Implementing BIM technologies in buildings can enhance the efficiency of construction projects. Adopting BIM technologies in developed countries contributes positive results in their national economy, but unfortunately, in emerging countries, especially in (KSA), BIM technologies are at an infant stage. The construction industry of (KSA) could gain different benefits and upsurge its performance by applying BIM technologies in construction projects. Four levels of BIM drivers were recognized after investigating BIM DFs through relevant literature, FDM, brainstorming sessions, and the ISM-MICMAC approach. The DFs enlisted at the bottom of the ISM model were considered significant drivers towards the adoption of BIM, but the elements placed at the top of the ISM model were considered least important.

Four hierarchical levels were identified through the ISM approach. Reduced cycle time of the design process DF13 and efficient construction planning and management DF1 were placed on the bottom level, which was deemed crucial. The reduced cycle time of the design process DF13 significantly increases efficiency and performance in the construction sector. Previously, traditional drawing methods were time-consuming, but BIM technology has streamlined project design. BIM integrates all project stakeholders, enabling more accessible review and approval of designs before construction begins. According to Gao, Guo [88], BIM ensures that all participants address their inquiries and ideas before the project starts. Efficient construction planning and management DF1 also play a significant role in the success of construction projects. Productive planning and management assist in saving time, reducing cost, and eliminating project failures. BIM technology encourages this by delivering a transparent and clear visualization of the project before it starts, facilitating better planning and management. Charef [89] analyzed that BIM assists construction practitioners in clearly understanding and managing their planning and implementation before the project starts.

A better understanding of energy consumption DF14, Reduction in labor work DF10, Visualization of data DF6, and Improved safety performance DF2 were considered major DFs at level three, demonstrating their greater significance than those at level two.

Understanding energy consumption DF14 is critical at this level because conventional energy consumption patterns enhance environmental degradation, depletion of resources, and energy shortages. The incorporation of BIM technology helps stakeholders focus on energy-saving strategies. Zakeri, Tabatabaee [1] identified that adopting cutting-edge technologies to manage energy more effectively can increase project efficiency. Reduction in labor work DF10 is another key driver. BIM facilitates design and project management through computer software, minimizing the requirement for human capital and thus enhancing work performance and financial gains for stakeholders. Visualization of data DF6 is also significant DF in level three. BIM technology offers a transparent view of project data, fostering stakeholder decision-making, and visible project visualization aids in anticipating and maintaining surprising incidents [36]. DF2 is also an imperative driver for improving safety performance because BIM technology encourages better safety measures by offering comprehensive safety information and increasing site safety performance.

Level two includes reducing document error and omission DF12, controlling whole-life costs and environmental data DF9, increasing sustainability DF8, enhancing collaboration among stakeholders DF7, and Improving quality DF4. The elements in level two are more significant than level one. Unfortunately, in the era of traditional drawing works, architectural work contains many mistakes and errors that cause failure in the project design; however, many difficulties and human errors have been overcome after the introduction of BIM technology. BIM technology assists stakeholders in minimizing the risk of error in paper documentation [90]. Controlled whole-life costs and environmental data DF9 is another potential driver enlisted in level two. Estimating the project life cycle cost is a challenging task during project design. Still, BIM technology significantly contributes to controlling the whole life cycle cost and providing a better understanding of the environmental issues at the earlier stage of projects. Increased sustainability DF8 is also an essential driver regarding the application of BIM technology in the construction industry because ecological pollution has been worsening worldwide in the last two decades. Most developed countries seriously consider minimizing carbon emissions, which is only possible through adopting sustainable technology. However, in developing countries, BIM can be a suitable approach to attain ecological development in building projects. Enhance collaboration among stakeholders DF7 is a critical driver that could help stakeholders promote BIM adoption in construction projects. BIM technology has brought different stakeholders under one umbrella, allowing them to share their ideas and experiences and coordinate with each other in the construction industry. The last and least important level two element is improving project quality. B4: Adopting BIM technology in project design helps enhance project quality, through which the assessment of construction resources and the work process can be judged easily.

Four potential drivers have been identified in level one: Reduce Risk DF15, Optimize the Owner’s experience and satisfaction DF11, reduce cost DF5, and Increase organization image DF3. Identifying the risk before the start of any project is challenging, but BIM technology assists stakeholders in eliminating the risk throughout the construction of buildings. Optimizing the Owner’s experience and satisfaction DF11 has been a significant potential driver for implementing BIM in building projects. The adoption of BIM provides internal satisfaction in the minds of stakeholders, which could also help expand the experience of project owners. Cost is another essential factor that catches the attention of stakeholders because, in recent innovations, BIM technology helps stakeholders minimize their projects cost-efficiently. The last but least crucial potential driver is increased organization image DF3. Organizations’ effective policies regarding applying BIM technologies during construction projects could help them gain a competitive edge in the market.

## 5. Conclusion

This study aimed to explore the drivers of BIM implementation from the perspective of the (KSA) construction industry. This pioneering study used the unique ISM-MICMAC approach to identify the significant DFs of BIM adoption in the construction industry of (KSA). The insights gained can enhance collaboration, improve project outcomes, increase efficiency, and promote sustainable practices within the industry.

The significant findings of this study show that Reduced cycle time of the design process DF13, Efficient construction planning and management DF1, Better understanding of energy consumption DF14, Reduction in labor work DF10, Visualization of data DF6, and Improved safety performance DF2 are essential potential drivers of BIM implementation in the construction industry. Furthermore, the construction industry of (KSA) is initially considering adopting BIM in building projects. The construction sector’s attention to applying BIM in building projects could encourage and motivate different stakeholders. The initial cost of BIM technology is high; however, the drivers of BIM technology adoption are long-lasting and could produce fruitful results and help to contribute to national economic growth.

### 5.1. Theoretical and practical implications

The study of BIM implementation of DF in the (KSA) through the ISM-MICMAC approach provides essential theoretical and practical implications. Theoretically, this study contributes to the awareness of how intricate relationships among different DFs can be systematically analyzed employing the ISM-MICMAC approach, providing a detailed framework for identifying BIM implementation dynamics in emerging markets. Practically, the outcomes of this study can guide policymakers, construction practitioners, and project managers in (KSA) by examining significant factors that influence BIM adoption, allowing them to tailor policies and interventions that advocate specific challenges and leverage drivers to increase the adoption process. This approach informs strategic decision-making and encourages the establishment of more productive policies and practices to enhance BIM integration in the (KSA) construction sector.

### 5.2. Limitations and future directions

Despite analyzing and evaluating DFs of BIM in construction projects of (KSA), there is still space to sort out and examine more DFs. This novel study left several questions for further research. So, further investigation could be done in the following context, like this research has been conducted from the (KSA) construction industry perspective. In the future, more investigations can be performed in other developing countries. The driving forces are selected with experts’ opinions, and these DFs are according to the existing situation of the construction sector of (KSA). In the future, experts’ views might be changed regarding that situation. The ISM methodology defines the complex relationship of the different DFs, so this methodology is not statistically proven. In the future, other methods, such as the structural equation modeling SEM approach, can be used to establish these DFs statistically. The research framework of this study has been developed according to the existing nature of variables; in the future, the nature of elements might be progressive, which could be meaningful for improving the research framework by applying the same methodologies.

## Supporting information

S1 Dataset Minimal data set. (DOCX)
