# Supplementary material for: Blueprint for progress: Understanding the driving forces of BIM adoption in Kingdom of Saudi Arabia (KSA) construction industry
Source: PLoS One. 2025 Feb 10;20(2):e0313135. doi: 10.1371/journal.pone.0313135 (PMC11809894; doi:10.1371/journal.pone.0313135)
Supplement: S1 Dataset — (DOCX) [file pone.0313135.s001.docx]

**Minimal data set**

| **Table A1. Literature search criteria** | |
| --- | --- |
| Keywords  (Level 1) | "BIM construction projects" OR 'Adoption of BIM" OR 'Benefits of BIM" OR" drivers of BIM" OR 'BIM advantages" OR" BIM Issues" OR" Role of BIM" OR" BIM technologies benefits in the construction industry" OR" BIM practices" OR "3D BIM" OR" 5D BIM" OR" 7D BIM" OR" sustainable BIM" OR" |
| Keywords  (Level 2) | "Government" OR" Rules and regulations" OR "sustainable BIM adoption" OR" innovative BIM technologies" OR "BIM in Pakistan construction industry" |
| Exclusion Criteria | A paper has the only title, author name, some keywords, and abstract; an article not focusing on review, survey, sound methodologies, or strong discussion; a paper not belonging to sustainability criteria. |

| Table A2. Experts' Ranking of drivers for BIM | | | | | | | | | | | |  |  |  |  |  |  |
| --- | --- | --- | --- | --- | --- | --- | --- | --- | --- | --- | --- | --- | --- | --- | --- | --- | --- |
| **Experts response** | | | | | | | | |  |  |  |  |  |  | **Sum** | **Mean** | **SD** |
| **No** | **Drivers** | **E1** | **E2** | **E3** | **E4** | **E5** | **E**6 | **E7** | **E8** | **E9** | **E10** | **E11** | **E12** | **E13** |  |  |  |
|  |  |  |  |  |  |  |  |  |  |  |  |  |  |  |  |  |  |
| 1 | Efficient construction planning and management | 4 | 5 | 5 | 4 | 5 | 4 | 5 | 5 | 4 | 4 | 5 | 5 | 3 | 58 | 4.4 | 0.66 |
| 2 | Improve safety performance | 5 | 5 | 4 | 4 | 5 | 4 | 5 | 4 | 5 | 5 | 5 | 5 | 4 | 60 | 4.6 | 0.50 |
| 3 | Increase organization image | 5 | 5 | 5 | 5 | 4 | 4 | 5 | 5 | 5 | 5 | 5 | 4 | 5 | 62 | 4.7 | 0.43 |
| 4 | Improve quality | 5 | 5 | 5 | 5 | 3 | 5 | 4 | 4 | 4 | 4 | 4 | 5 | 5 | 58 | 4.4 | 0.66 |
| 5 | Reduce cost | 4 | 4 | 5 | 4 | 5 | 5 | 5 | 5 | 5 | 5 | 5 | 5 | 5 | 62 | 4.7 | 0.4 |
| 6 | Visualization of data | 5 | 4 | 5 | 5 | 5 | 5 | 5 | 4 | 5 | 4 | 5 | 5 | 4 | 61 | 4.6 | 0.48 |
| 7 | Enhance collaboration among stakeholders | 5 | 5 | 5 | 5 | 4 | 5 | 5 | 4 | 5 | 5 | 5 | 5 | 4 | 62 | 4.7 | 0.43 |
| 8 | Increase sustainability | 5 | 5 | 5 | 4 | 5 | 4 | 4 | 4 | 4 | 4 | 4 | 4 | 4 | 56 | 4.3 | 0.48 |
| 9 | Controlled whole-life costs and environmental data | 5 | 5 | 5 | 5 | 5 | 5 | 3 | 4 | 5 | 5 | 5 | 4 | 5 | 61 | 4.6 | 0.63 |
| 10 | Reduction in labor work | 5 | 5 | 5 | 5 | 5 | 5 | 4 | 5 | 5 | 5 | 5 | 5 | 5 | 64 | 4.9 | 0.27 |
| 11 | Optimize the Owner's experience and satisfaction | 5 | 5 | 4 | 5 | 5 | 4 | 4 | 4 | 4 | 4 | 5 | 5 | 5 | 57 | 4.5 | 0.51 |
| 12 | Reduction in documents error and omission | 5 | 5 | 5 | 5 | 5 | 3 | 4 | 4 | 5 | 5 | 5 | 4 | 5 | 60 | 4.6 | 0.65 |
| 13 | Reduced cycle time of the design process | 5 | 4 | 4 | 5 | 5 | 5 | 5 | 4 | 4 | 4 | 4 | 5 | 5 | 59 | 4.5 | 0.51 |
| 14 | Better understanding of energy consumption | 5 | 5 | 5 | 5 | 5 | 5 | 3 | 5 | 5 | 4 | 4 | 5 | 5 | 61 | 4.6 | 0.63 |
| 15 | Reduce Risk | 4 | 4 | 5 | 5 | 5 | 5 | 4 | 5 | 5 | 5 | 4 | 4 | 4 | 59 | 4.5 | 0.51 |

| **Table A3. Comparison of ISM-MICMAC with different MCDM techniques** | | | | |
| --- | --- | --- | --- | --- |
| **ISM-MICMAC** | **Analytical hierarchy process (AHP)** | **Structural equation modeling (SEM)** | **DEMATEL** | **Analytical network process (ANP)** |
| ISM shows the contextual correlation between factors depending on dependencies and driving powers. | AHP does not propose interdependencies  among variables. AHP is accustomed to displaying a hierarchical model of elements | SEM is a basic statistical approach. SEM studies need a collection of data and extensive sampling to a theoretical framework | DEMATL approach affirms, the coincidental-effects  links, among variables | ANP is a complex approach that is no more widely adopted; it proposed independencies among variables. |

| **Table A4. Experts profile** | | | | | | |
| --- | --- | --- | --- | --- | --- | --- |
| **No** | **Types of business** | **Qualification** | **Designation** | **Relevant expertise** | **Experience** | **Firm size** |
| 1 | Consultant | Bachelor (Project Management) | Manager | Expert in business consulting; | 10 | 25 |
| 2 | Consultant | Bachelor (Project Management) | Director | Specialized in strategic consulting and business development | 13 | 38 |
| 3 | Consultant | Master (Disaster Management) | Director | Expert in business process optimization and change management | 11 | 25 |
| 4 | Civil Engineer | Bachelor (Civil Engineering) | Director | Expert in infrastructure project management | 25 | 40 |
| 5 | Civil Engineer | Master (Construction Engineering and Management) | Associate engineer | Specialist in structural analysis and civil engineering design | 14 | 110 |
| 6 | Architect | Master (Urban Design and Planning) | Project manager | Experienced in architectural design and project management. | 9 | 200 |
| 7 | Architect | Master (landscape architecture) | Project architect | Specializes in sustainable architecture and project design | 17 | 30 |
| 8 | Architect | Master (Industrial architecture) | Project architect | Focus on architectural innovation and design. | 14 | 22 |
| 9 | Architect | Master (Industrial Architecture) | Associate architect | Expert in residential and commercial architecture. | 8 | 10 |
| 10 | Institution | PhD (Infrastructural Engineering) | Professor | Expert with a focus on advanced materials and nanotechnology | 13 | 2500 |
| 11 | Institution | PhD (Environmental Engineering) | Professor | Expert in renewable energy systems and sustainable engineering. | 16 | 2500 |
| 12 | Institution | Postdoc (Architecture) | professor | Specialist in digital architecture and 3D modeling technologies. | 15 | 4000 |
| 13 | Institution | PhD (Project Management) | Associate professor | Expert in strategic management and leadership development. | 8 | 4500 |

| **Table A5. initial reachability matrix** | | | | | | | | | | | | | | | |
| --- | --- | --- | --- | --- | --- | --- | --- | --- | --- | --- | --- | --- | --- | --- | --- |
| **Sr. No** | 1 | 2 | 3 | 4 | 5 | 6 | 7 | 8 | 9 | 10 | 11 | 12 | 13 | 14 | 15 |
| 1 | 1 | 1 | 1 | 1 | 1 | 1 | 1 | 1 | 1 | 1 | 1 | 1 | 1 | 1 | 1 |
| 2 | 0 | 1 | 1 | 1 | 1 | 1 | 0 | 1 | 0 | 1 | 1 | 0 | 0 | 1 | 1 |
| 3 | 0 | 0 | 1 | 0 | 0 | 0 | 0 | 0 | 0 | 0 | 0 | 0 | 0 | 1 | 1 |
| 4 | 0 | 1 | 1 | 1 | 0 | 0 | 0 | 1 | 1 | 0 | 1 | 1 | 1 | 1 | 1 |
| 5 | 0 | 0 | 1 | 0 | 1 | 0 | 0 | 0 | 0 | 0 | 0 | 0 | 0 | 0 | 1 |
| 6 | 1 | 1 | 0 | 1 | 1 | 1 | 1 | 0 | 1 | 0 | 1 | 1 | 1 | 1 | 1 |
| 7 | 0 | 1 | 1 | 1 | 1 | 0 | 1 | 1 | 1 | 1 | 1 | 1 | 1 | 1 | 1 |
| 8 | 0 | 0 | 1 | 0 | 0 | 0 | 1 | 1 | 0 | 0 | 1 | 0 | 0 | 1 | 1 |
| 9 | 0 | 1 | 1 | 0 | 1 | 1 | 0 | 1 | 1 | 0 | 1 | 0 | 0 | 0 | 0 |
| 10 | 1 | 0 | 0 | 1 | 1 | 0 | 0 | 1 | 1 | 1 | 0 | 0 | 0 | 0 | 0 |
| 11 | 0 | 0 | 1 | 1 | 1 | 0 | 1 | 0 | 0 | 0 | 1 | 0 | 0 | 0 | 0 |
| 12 | 0 | 0 | 1 | 0 | 1 | 0 | 0 | 1 | 0 | 0 | 1 | 1 | 0 | 0 | 1 |
| 13 | 0 | 0 | 1 | 0 | 1 | 0 | 0 | 1 | 1 | 1 | 1 | 1 | 1 | 0 | 0 |
| 14 | 0 | 1 | 0 | 1 | 0 | 0 | 0 | 0 | 1 | 0 | 1 | 1 | 0 | 1 | 1 |
| 15 | 0 | 0 | 1 | 0 | 1 | 0 | 0 | 0 | 0 | 1 | 1 | 0 | 0 | 0 | 1 |

| **Table A6. Final reachability matrix** | | | | | | | | | | | | | | | | |
| --- | --- | --- | --- | --- | --- | --- | --- | --- | --- | --- | --- | --- | --- | --- | --- | --- |
| **Sr.**  **No** | 1 | 2 | 3 | 4 | 5 | 6 | 7 | 8 | 9 | 10 | 11 | 12 | 13 | 14 | 15 | **DR.**  **power** |
| 1 | 1 | 1 | 1 | 1 | 1 | 1 | 1 | 1 | 1 | 1 | 1 | 1 | 1 | 1 | 1 | 15 |
| 2 | 1* | 1 | 1 | 1 | 1 | 1 | 1* | 1 | 1* | 1 | 1 | 1* | 1* | 1 | 1 | 15 |
| 3 | 0 | 1* | 1 | 1* | 1* | 0 | 0 | 0 | 1* | 1* | 1* | 1* | 0 | 1 | 1 | 10 |
| 4 | 0 | 1 | 1 | 1 | 1* | 1* | 1* | 1 | 1 | 1* | 1 | 1 | 1 | 1 | 1 | 14 |
| 5 | 0 | 0 | 1 | 0 | 1 | 0 | 0 | 0 | 0 | 1* | 1* | 0 | 0 | 0 | 1 | 5 |
| 6 | 1 | 1 | 1* | 1 | 1 | 1 | 1 | 1* | 1 | 1* | 1 | 1 | 1 | 1 | 1 | 15 |
| 7 | 1* | 1 | 1 | 1 | 1 | 1* | 1 | 1 | 1 | 1 | 1 | 1 | 1 | 1 | 1 | 15 |
| 8 | 0 | 1* | 1 | 1* | 1* | 0 | 1 | 1 | 1* | 1* | 1 | 1* | 0 | 1 | 1 | 12 |
| 9 | 0 | 1 | 1 | 1* | 1 | 1 | 1* | 1 | 1 | 0 | 1 | 0 | 0 | 0 | 0 | 9 |
| 10 | 1 | 1* | 1* | 1 | 1 | 1* | 1* | 1 | 1 | 1 | 1* | 1* | 1* | 1* | 1* | 15 |
| 11 | 1* | 1* | 1 | 1 | 1 | 0 | 1 | 1* | 1* | 1* | 1 | 1* | 1* | 1* | 1* | 14 |
| 12 | 0 | 0 | 1 | 1* | 1 | 0 | 1* | 1 | 0 | 1* | 1 | 1 | 0 | 1* | 1 | 10 |
| 13 | 1* | 1* | 1 | 1* | 1 | 1* | 1* | 1 | 1 | 1 | 1 | 1 | 1 | 1* | 1* | 15 |
| 14 | 0 | 1 | 1* | 1 | 1* | 1* | 1* | 1* | 1 | 1* | 1 | 1 | 0 | 1 | 1 | 14 |
| 15 | 1* | 0 | 1 | 1* | 1 | 0 | 1* | 0 | 0 | 1 | 1 | 0 | 0 | 0 | 1 | 8 |
| **Dep. power** | 8 | 12 | 15 | 14 | 15 | 9 | 13 | 12 | 12 | 14 | 15 | 12 | 9 | 12 | 14 | **186** |

| **Table A7. Iteration Levels** | | | | |
| --- | --- | --- | --- | --- |
| **Sr# Reachability set Antecedent set Intersection set Level** | | | | |
| **1** | 2,3,4,5,9,10,11,12,14,15 | 1,2,3,4,5,6,7,8,9,10,11,12,13,14,15 | 2,3,4,5,9,10,11,12,14,15 | **IV** |
| **2** | 3,5,10,11,15 | 1,2,3,4,5,6,7,8,9,10,11,12,13,14,15 | 3,5,10,11,15 | **III** |
| **3** | 1,2,3,4,5,7,8,9,10,11,12,13,14,15 | 1,2,3,4,5,6,7,8,9,10,11,12,13,14,15 | 1,2,3,4,5,7,8,9,10,11,12,13,14,15 | **I** |
| **4** | 1,3,4,5,7,10,11,15 | 1,2,3,4,5,6,7,8,10,11,12,13,14,15 | 1,3,4,5,7,10,11,15 | **II** |
| **5** | 2,4,6,7,8,9,10,12,13,14 | 1,2,4,6,7,8,9,10,12,13,14 | 2,4,6,7,8,9,10,12,13,14 | **I** |
| **6** | 1,2,4,6,7,8,9,10,12,13,14 | 1,2,4,6,7,8,9,10,12,13,14 | 1,2,4,6,7,8,9,10,12,13,14 | **III** |
| **7** | 2,4,7,8,9,10,12,14 | 1,2,4,6,7,8,9,10,12,13,14 | 2,4,7,8,9,10,12,14 | **II** |
| **8** | 2,4,6,7,8,9 | 1,2,4,6,7,8,9,10,13,14 | 2,4,6,7,8,9 | **II** |
| **9** | 4,7,8,10,12,14 | 1,2,4,6,7,8,10,12,13,14 | 4,7,8,10,12,14 | **II** |
| **10** | 1,2,6,10,13,14 | 1,2,6,10,13,14 | 1,2,6,10,13,14 | **III** |
| **11** | 1,2,6,10,13,14 | 1,2,6,9,10,13,14 | 1,2,6,10,13,14 | **I** |
| **12** | 1,2,6,10,13,14 | 1,2,6,8,10,12,13,14 | 1,2,6,10,13,14 | **II** |
| **13** | 2,6,10,13,14 | 1,2,6,10,13,14 | 2,6,10,13,14 | **IV** |
| **14** | 1,13 | 1,13 | 1,13 | **III** |
| **15** | 1,13 | 1,13 | 1,13 | **I** |

| **Table A8. Hierarchical levels of BIM drivers** | | |
| --- | --- | --- |
| **No.** | **BIM Drivers** | **Levels** |
| 3 | Increase organization image | I |
| 5 | Reduce cost | I |
| 11 | Optimize the Owner's experience and  satisfaction | I |
| 15 | Reduce Risk | I |
| 4 | Improve Project quality | II |
| 7 | Enhance collaboration among stakeholders | II |
| 8 | Increase sustainability | II |
| 9 | Controlled whole-life costs and environmental data | II |
| 12 | Reduction in documents error and omission | II |
| 2 | Improve safety performance | III |
| 6 | Visualization of data | III |
| 10 | Reduction in labor work | III |
| 14 | A better understanding of energy consumption | III |
| 1 | Efficient construction planning and management | IV |
| 13 | Reduced cycle time of the design process | IV |
